# Serum Zinc Level and non-Protein Respiratory Quotient in Patients with Chronic Liver Diseases

**DOI:** 10.3390/jcm9010255

**Published:** 2020-01-17

**Authors:** Hiroki Nishikawa, Ryo Takata, Hirayuki Enomoto, Kazunori Yoh, Yoshinori Iwata, Yoshiyuki Sakai, Kyohei Kishino, Yoshihiro Shimono, Naoto Ikeda, Tomoyuki Takashima, Nobuhiro Aizawa, Kunihiro Hasegawa, Noriko Ishii, Yukihisa Yuri, Takashi Nishimura, Hiroko Iijima, Shuhei Nishiguchi

**Affiliations:** Division of Hepatobiliary and Pancreatic disease, Department of Internal Medicine, Hyogo College of Medicine, Nishinomiya, Hyogo 663-8501, Japan; chano_chano_rt@yahoo.co.jp (R.T.); enomoto@hyo-med.ac.jp (H.E.); mm2wintwin@ybb.ne.jp (K.Y.); yo-iwata@hyo-med.ac.jp (Y.I.); sakai429@hyo-med.ac.jp (Y.S.); hcm.kyohei@gmail.com (K.K.); yoshihiro19870729@yahoo.co.jp (Y.S.); nikeneko@hyo-med.ac.jp (N.I.); tomo0204@yahoo.co.jp (T.T.); nobu23hiro@yahoo.co.jp (N.A.); hiro.red1230@gmail.com (K.H.); ishinori1985@yahoo.co.jp (N.I.); gyma27ijo04td@gmail.com (Y.Y.); tk-nishimura@hyo-med.ac.jp (T.N.); hiroko-i@hyo-med.ac.jp (H.I.); nishiguc@hyo-med.ac.jp (S.N.)

**Keywords:** chronic liver disease, non-protein respiratory quotient, zinc, indirect calorimetry, correlation

## Abstract

We sought to clarify the correlation between non-protein respiratory quotient (npRQ) in indirect calorimetry and serum zinc (Zn) level in chronic liver diseases (CLDs, *n* = 586, 309 liver cirrhosis (LC) patients, median age = 63 years). Clinical parameters potentially linked to npRQ <0.85 (best cutoff point for the prognosis in LC patients) were also examined in receiver operating characteristic curve (ROC) analyses. The median npRQ was 0.86. The median serum Zn level was 64 μg/dL. The median npRQ in patients with non-LC, Child–Pugh A, Child–Pugh B and Child–Pugh C were 0.89, 0.85, 0.83 and 0.82 (overall *p* < 0.0001)). The median serum Zn level in patients with npRQ <0.85 (58 μg/dL) was significantly lower than that in patients with npRQ ≥ 0.85 (68 μg/dL) (*p* < 0.0001). The correlation coefficient (*r*) between npRQ level and serum Zn level for all cases was 0.40 (*p* < 0.0001). Similar tendencies were observed in all subgroup analyses. The highest correlation coefficient between serum Zn level and npRQ was found in patients with Child–Pugh C (*n* = 22, *r* = 0.69). In ROC analyses for npRQ <0.85, serum Zn level had the highest area under the ROC (AUC) among baseline laboratory parameters (AUC = 0.69). In conclusion, serum Zn level can be helpful for npRQ in patients with CLDs.

## 1. Introduction

The liver is a key player for the metabolism and preservation of nutrients, and its deep involvement can be seen in the maintenance of serum blood glucose levels or gluconeogenesis from glycogen, etc [1,2,3]. Liver cirrhosis (LC), which can develop due to persistent inflammation over a long period of time in the liver, is frequently accompanied by protein-energy malnutrition (PEM) [1,2,4,5,6,7]. Assessment methods for protein malnutrition and energy malnutrition are in general serum albumin level and the non-protein respiratory quotient (npRQ) in indirect calorimetry (IC, metabolic cart) [8]. RQs suggest which macronutrients (i.e., carbohydrates, protein or fat) are being metabolized: RQ value which approaches 1.0 denotes that carbohydrates are mainly being consumed as an energy source and RQ value which approaches 0.7 denotes that fats are being consumed as an energy source [9]. RQ value can exceed 1.00 during hard exercise [9]. npRQ indicates RQ excluding protein burning [8]. PEM is a common disorder identified in LC patients, which can cause worse clinical outcomes in LC patients [1,2,4,5,6,8,10,11]. In our previous study, we demonstrated the significant correlation between npRQ level and albumin-bilirubin (ALBI) grade, which can be an alternative classification system for Child–Pugh classification, in patients with chronic liver diseases (CLDs) [12,13].

Zinc (Zn) is widely distributed in the human body, being a key trace element for normal cell growth and normal cell differentiation [14]. Zn is essential for the catalytic activity of numerous enzymes involved in energy nutrition metabolism and seems to regulate several hormones which play important roles in the metabolism [15]. Zn deficiency is a major health issue worldwide and causes numerous clinical symptoms [16,17,18,19]. The homeostasis of Zn is primarily regulated in the liver and the degree of Zn loss in CLD patients has been shown to be well correlated with the liver diseases severity [20,21,22,23,24,25]. In our recent reports, we have shown the close relationship between serum Zn level and sarcopenia as defined by low muscle strength and low skeletal muscle decline in CLD patients [26]. Sarcopenia in CLDs can be linked to the liver diseases severity [27]. However, the relationship between npRQ level in IC and serum Zn level in CLD patients has been largely unknown. To elucidate the issue seems to be clinically of importance because IC is expensive and involves time-consuming procedures, while serum Zn level can be obtained easily in the clinical settings [28,29]. In the current study, we sought to clarify this important research question.

## 2. Patients and Methods

### 2.1. Patients

A total of 601 CLD patients with data for npRQ in IC available were admitted to our hospital between October 2005 and August 2018. Of these, 15 patients had missing data for serum Zn level. A total of 586 CLD patients having data for both npRQ in IC and serum Zn level were thus subjected to the current analysis. Liver histological data (F0–F4) were available in all analyzed patients and bioimpedance analysis (BIA) data were available in 580 patients (99.0%).

### 2.2. Measurement of npRQ by IC

Oxygen consumption in one minute (VO_2_) and carbon dioxide production in one minute (VCO_2_) were tested by IC. As explained in the introduction section, npRQ indicates RQ excluding protein burning. Total urinary nitrogen excretion (UN) was measured as reported elsewhere [8,30]. npRQ was calculated by the formula as reported previously [6,25].

### 2.3. SMI and ECW to TBW Ratio using BIA

The definition of skeletal muscle index (SMI) is “appendicular skeletal muscle mass (SMM) divided by height squared (kg/m^2^)” in BIA. Referring to the current Japanese criteria, the definition of SMM loss was: SMI < 7.0 kg/m^2^ in men and <5.7 kg/m^2^ in women [27]. Considering the fact that excessive extracellular water (ECW) results in edematous condition, extracellular fluid (ECF) status was considered to correspond to the ECW to total body water (TBW) ratio (ECW/TBW). In healthy individuals, ECW/TBW is able to be kept at a constant level (around 0.38). ECF excess was categorized into the following three conditions referring to ECW/TBW: normal condition (<0.390), mild overhydrated condition (0.390–0.399) and moderate to severe overhydrated condition (≥0.400) [31].

The relationship between npRQ and serum Zn level, and clinical parameters potentially linked to npRQ < 0.85 (best cutoff value for clinical outcomes in LC patients) was studied in receiver operating characteristic curve (ROC) analyses [8,28,32]. This study was done per the ethical principles of the 1975 Declaration of Helsinki and was approved by the institutional review board in Hyogo college of medicine hospital (approval no.1831).

### 2.4. Statistical Considerations

In the analysis of continuous variables, for the purpose of assessing between-group difference, Student’s *t* test, Mann–Whitney *U* test, Pearson’s correlation coefficient *r*, analysis of variance or Kruskal-Wallis test were in employment as applicable. ROC analysis was done for calculating the area under the ROC (AUC) for laboratory parameters choosing the optimal cutoff value that maximized the total value of sensitivity and specificity for npRQ <0.85. Clinical data were shown as median value (interquartile range (IQR)). A *p* < 0.05 was considered statistical significance. The JMP 14 (SAS Institute Inc., Cary, NC) was in employment for statistical analysis.

## 3. Results

### 3.1. Patient Baseline Backgrounds

**Figure 1:**
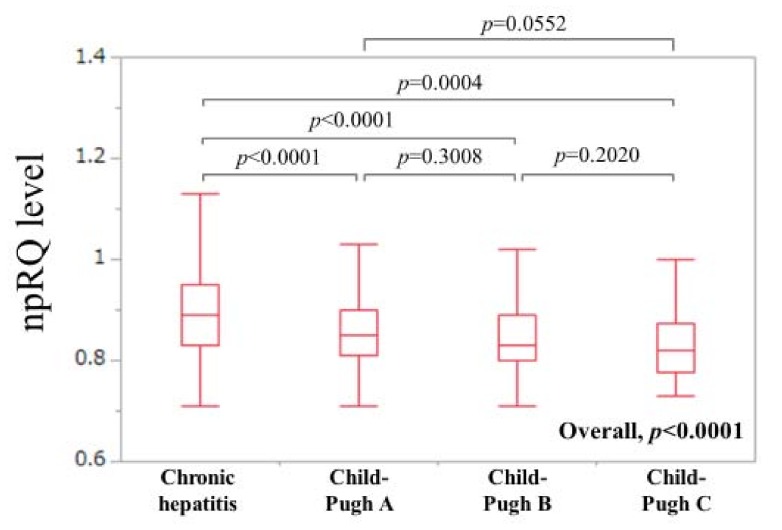
Comparison of npRQ (non-protein respiratory quotient) level in patients with chronic hepatitis (non-LC; non-liver cirrhosis), Child–Pugh A, Child–Pugh B and Child–Pugh C.

Patient baseline backgrounds in the study (*n* = 586, 290 men and 296 women, median (IQR) age = 63 (54,71) years) are shown in Table 1. The median (IQR) npRQ was 0.86 (0.82, 0.93). Two hundred and forty-two patients (41.3%) had npRQ <0.85. The median (IQR) serum Zn level was 64 μg/dL (55, 73 μg/dL). In terms of cause for liver disease, 309 patients (52.7%) had hepatitis C virus (HCV). In terms of the severity of liver fibrosis (F factor), there were 12 patients in F0, 113 in F1, 77 in F2, 75 in F3 and 309 in F4. In LC patients (*n* = 309), Child–Pugh A was found in 169 patients, Child–Pugh B in 118 and Child–Pugh C in 22. The median (IQR) npRQ in patients with chronic hepatitis (non-LC), Child–Pugh A, Child–Pugh B and Child–Pugh C were: 0.89 (0.83, 0.95), 0.85 (0.81, 0.9), 0.83 (0.80, 0.89) and 0.82 (0.7775, 0.8725) (*p* values: CH versus Child–Pugh A, *p* < 0.0001; Child–Pugh A versus Child–Pugh B, *p* = 0.3008; Child–Pugh B versus Child–Pugh C, *p* = 0.2020; CH versus Child–Pugh B, *p* < 0.0001; CH versus Child–Pugh C, *p* < 0.0001; Child–Pugh A versus Child–Pugh C, *p* = 0.0552; (overall *p* < 0.0001)). (Figure 1) Hepatocellular carcinoma (HCC) was found in 161 patients (27.5%). Ascites was identified in 50 patients (8.5%). The median (IQR) npRQ in patients with HCC and without HCC were: 0.84 (0.80, 0.90) and 0.87 (0.82, 0.94) (*p* = 0.0074). The median (IQR) npRQ in patients with ascites and without ascites were: 0.82 (0.78, 0.85) and 0.87 (0.82, 0.93) (*p* < 0.0001). 

### 3.2. Correlation between npRQ Level and Serum Zn Level for All Cases

**Figure 2:**
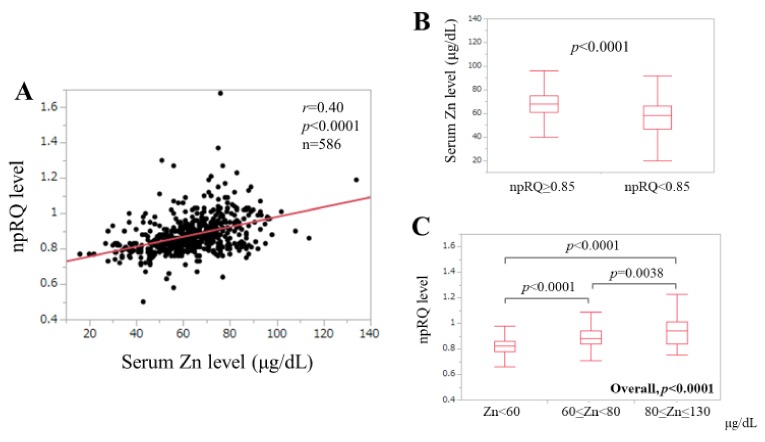
(**A**) Correlation between serum Zn level and npRQ (non-protein respiratory quotient) for all cases (*n* = 586). (**B**) Comparison of serum Zn level stratified by npRQ level. (**C**) Comparison of npRQ level based on the serum Zn classification system proposed by the Japanese society of clinical nutrition.

The correlation coefficient (*r*) between npRQ level and serum Zn level was 0.40 (*p* < 0.0001). (Figure 2A) The median (IQR) serum Zn level in patients with npRQ < 0.85 (*n* = 242, 58 μg/dL (47, 66.25 μg/dL)) was significantly lower than that in patients with npRQ ≥ 0.85 (*n* = 344, 68 μg/dL (61, 75 μg/dL)) (*p* < 0.0001). (Figure 2B) Currently, the Japanese society of clinical nutrition (JSCN) defines the followings: (A) serum Zn level <60 μg/dL corresponds to Zn deficiency; (B) 60 μg/dL ≤ serum Zn level < 80 μg/dL corresponds to subclinical Zn deficiency; and (C) 80 μg/dL ≤ serum Zn level < 130 μg/dL corresponds to normal Zn level [33]. The median npRQ levels in patients with Zn deficiency (*n* = 211), subclinical Zn deficiency (*n* = 305) and normal Zn range (*n* = 70) were: 0.82 (0.78, 0.86), 0.88 (0.84, 0.95) and 0.94 (0.8375, 1.01) (*p* < values: Zn deficiency versus subclinical Zn deficiency, *p* < 0.0001; subclinical Zn deficiency versus normal Zn level, *p* = 0.0038; Zn deficiency versus normal Zn level, *p* < 0.0001; overall *p* < 0.0001) (Figure 2C).

### 3.3. Correlation npRQ Level and Serum Zn Level According to the LC Status and the Child–Pugh Classification

**Figure 3:**
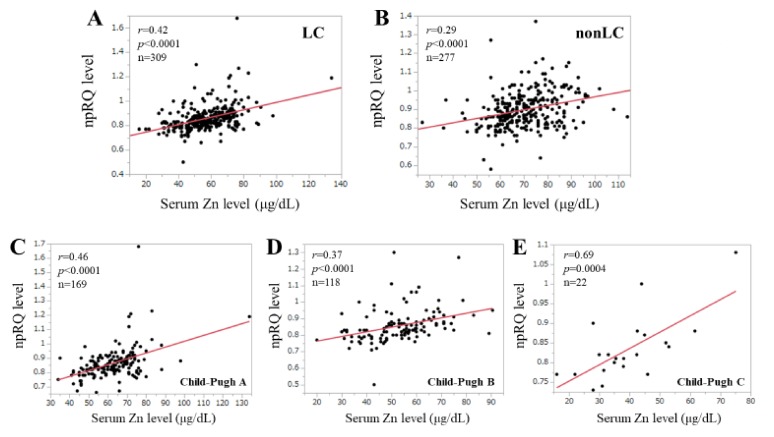
(**A**,**B**) Correlation between serum Zn level and npRQ (non-protein respiratory quotient) in patients with LC (liver cirrhosis) and non-LC (non-liver cirrhosis). (**C**–**E**) Correlation between serum Zn level and npRQ in patients with Child–Pugh (**A**–**C**).

In LC patients, there was a significant correlation between serum Zn level and npRQ level (*r* = 0.42, *p* < 0.0001) (Figure 3A). In non-LC patients, there was a significant correlation between serum Zn level and npRQ level (*r* = 0.29, *p* < 0.0001) (Figure 3B).

The median (IQR) serum Zn levels in patients with Child–Pugh A, B and C were 66 μg/dL (57.5, 72 μg/dL), 54 μg/dL (43.75, 60.4) μg/dL) and 38.05 μg/dL (30.75, 47.5) μg/dL). In Child–Pugh A, B and C patients, significant correlation was found between npRQ level and serum Zn level: Child–Pugh A, *r* = 0.46, *p* < 0.0001; Child–Pugh B, *r* = 0.37, *p* < 0.0001; Child–Pugh C, *r* = 0.69, *p* = 0.0004 (Figure 3C–E).

### 3.4. Correlation npRQ Level and Serum Zn Level According to the Body Mass Index (BMI) Value

**Figure 4:**
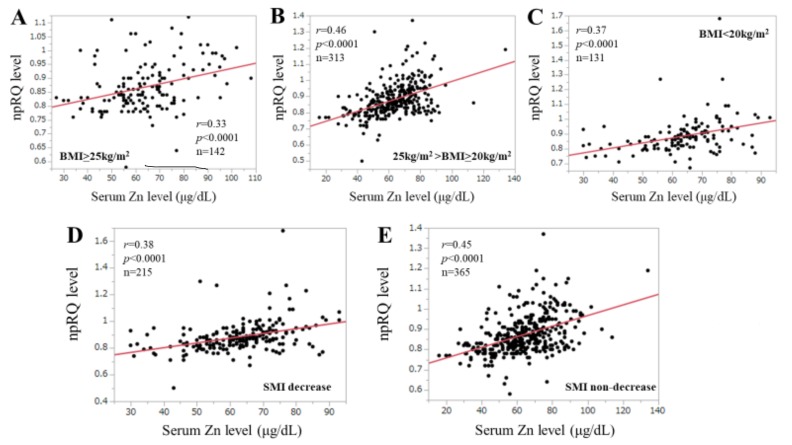
(**A**–**C**) Correlation between serum Zn level and npRQ according to the BMI status. (**D**,**E**) Correlation between serum Zn level and npRQ in patients with SMI decrease and SMI non-decrease.

The study population was divided into three groups based on the BMI value (*n* = 1): BMI ≥ 25 kg/m^2^ (*n* = 142), 25 kg/m^2^ > BMI ≥ 25 kg/m^2^ (*n* = 313) and BMI < 20 kg/m^2^ (*n* = 130). The correlation coefficients between npRQ level and serum Zn level in patients with BMI ≥ 25 kg/m^2^, 25 kg/m^2^ > BMI ≥ 25 kg/m^2^ and BMI < 20 kg/m^2^ were: *r* = 0.33 (*p* < 0.0001), *r* = 0.46 (*p* < 0.0001) and *r* = 0.37 (*p* < 0.0001) (Figure 4A–C).

### 3.5. Correlation npRQ Level and Serum Zn Level According to the SMI Value

The study population was divided into two groups based on the SMI value (*n* = 6): SMI decrease (*n* = 215) and SMI non-decrease (*n* = 365) using the Japanese guidelines [27]. The correlation coefficients between npRQ level and serum Zn level in patients with SMI decrease and SMI non-decrease were: *r* = 0.38 (*p* < 0.0001) and *r* = 0.45 (*p* < 0.0001) (Figure 4D,E).

### 3.6. Correlation npRQ Level and Serum Zn Level According to the ECW/TBW

**Figure 5:**
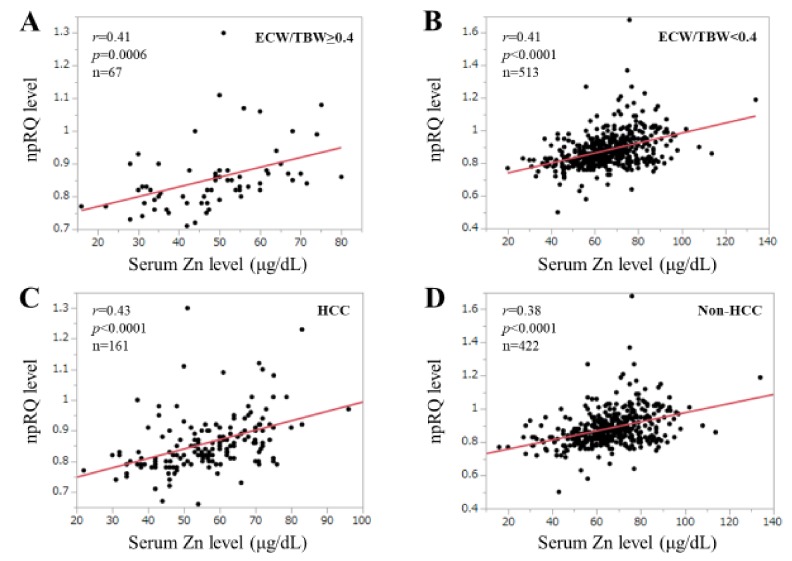
(**A**,**B**) Correlation between serum Zn level and npRQ (non-protein respiratory quotient) according to the ECW to TBW status. (**C**,**D**) Correlation between serum Zn level and npRQ in patients with HCC (Hepatocellular carcinoma) or without HCC.

The study population was divided into two groups based on the ECW/TBW (*n* = 6): ECW/TBW ≥ 0.40 (*n* = 67) and ECW/TBW < 0.40 (*n* = 513). The correlation coefficients between npRQ level and serum Zn level in patients with ECW/TBW ≥ 0.40 and ECW/TBW < 0.40 were: *r* = 0.41 (*p* = 0.0006) and *r* = 0.41 (*p* < 0.0001) (Figure 5A,B).

### 3.7. Correlation npRQ Level and Serum Zn Level According to the Presence or Absence of HCC

The correlation coefficients between npRQ level and serum Zn level in patients with HCC and without HCC were: *r* = 0.43 (*p* < 0.0001) and *r* = 0.38 (*p* < 0.0001) (Figure 5C,D).

### 3.8. Correlation npRQ Level and Serum Zn Level According to the Liver Disease Etiologies

**Figure 6:**
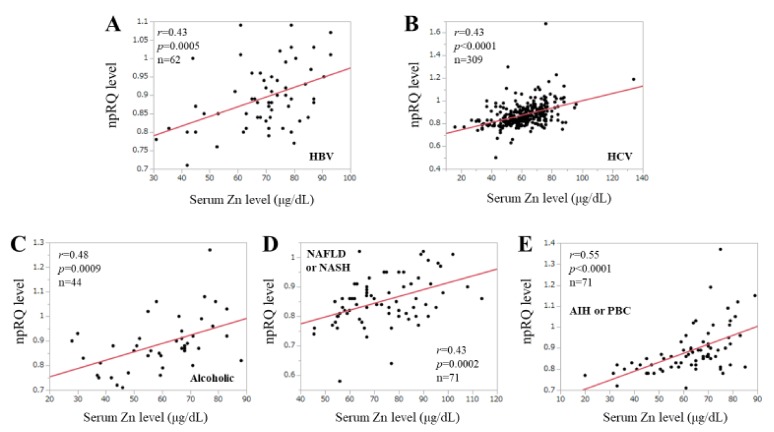
(**A**–**E**) Correlation between serum Zn level and npRQ (non-protein respiratory quotient) according to liver disease etiologies.

The correlation coefficients between npRQ level and serum Zn level in patients with hepatitis B virus (*n* = 62), HCV (*n* = 309), alcohol (*n* = 44), non-alcoholic fatty liver disease or non-alcoholic steatohepatitis (*n* = 71), and autoimmune hepatitis (AIH) or primary biliary cholangitis (PBC) (*n* = 71) were: *r* = 0.43 (*p* = 0.0005), *r* = 0.43 (*p* < 0.0001), *r* = 0.48 (*p* = 0.0009), *r* = 0.43 (*p* = 0.0002), and *r* = 0.55 (*p* < 0.0001) (Figure 6A–E).

### 3.9. ROC Analyses for the npRQ < 0.85

Results of ROC analysis for the npRQ < 0.85 in baseline laboratory parameters are presented in Table 2. Serum Zn level had the highest AUC (0.69), followed by prothrombin time (AUC = 0.65). The AUCs, optimal cutoff points, sensitivity (%) and specificity (%) in each parameter are shown in Table 2.

## 4. Discussion

The npRQ calculated from IC shows the ratio of carbohydrate to fat oxidation after excluding protein burning, and a lower npRQ value is shown to be an adverse predictor in LC patients [8]. However, as described earlier, IC is expensive and time-consuming [28,29]. Few clinicians are familiar with IC and thus it is not widely used in the clinical settings. Identifying markers linked to npRQ level in patients with CLDs appears to be clinically relevant, while serum Zn level can be obtained easily in the clinical settings. Zn plays an important role in various physiological functions. The current analysis was therefore done. The major strength of the study was its large sample number (*n* = 586). The current study is, to our knowledge, the first study of its kind studying the relationship between npRQ and serum Zn level in CLD patients.

In our results, npRQ level was significantly correlated with serum Zn level for all cases, and these tendencies were confirmed for all subgroup analyses. Additionally, serum Zn level had the highest AUC for predicting npRQ < 0.85, and npRQ level was well stratified by the current Zn classification system proposed by JSCN. These results showed that serum Zn level can be helpful for assessing energy metabolism in CLDs and Zn supplementation therapy for CLD patients with decreased serum Zn level may improve energy metabolism. An optimal supply of macro and micronutrients is the mainstay of nutritional therapy in CLD patients. A recent meta-analysis demonstrated the impact of a late evening snack on energy metabolism in LC patients [34].

As described in the introduction section, Zn is essential for the catalytic activity of numerous enzymes involved in energy nutrition metabolism [15]. Decreased serum Zn level can lead to the decrease of the catalytic activity of numerous enzymes, eventually resulting in a poorer energy nutritional status (i.e., poorer npRQ). In this study, this phenomenon is more prominent in patients with Child–Pugh C as shown in Figure 3E. In our data, the correlation coefficient between npRQ and serum Zn level in patients with Child–Pugh C was 0.69, which was the highest value in our analysis. In our Child–Pugh C patients (*n* = 22), 14 patients (63.6%) had npRQ < 0.85 and 20 patients (90.9%) had serum Zn level < 60 μg/dL. Most Child–Pugh C patients can have experience of hepatic events such as ascites or hepatic encephalopathy which consume a lot of energy [3,4,6]. Especially in patients with far advanced LC status, serum Zn level can be helpful for predicting npRQ. While in our subgroup analyses stratified by liver disease etiology, the strongest link between npRQ and serum Zn level was observed in patients with AIH or PBC (*r* = 0.55, *p* < 0.0001). AIH or PBC patients can present higher plasma levels of lactate, acetate, acetoacetate, pyruvate and glucose which are associated with energy metabolism alterations [35,36]. These observations may be related to our results.

The correlation coefficient between npRQ and serum Zn level in non-LC patients (*r* = 0.29) was lower than that in LC patients (*r* = 0.42) in this study. In non-LC patients, 84 patients (30.3%) had npRQ < 0.85 and 43 patients (15.5%) had serum Zn level < 60 μg/dL, while in LC patients, 158 patients (51.1%) had npRQ < 0.85 and 169 patients (54.7%) had serum Zn level < 60 μg/dL. Close linkage between npRQ and serum Zn level was found with the liver disease progression.

Energy metabolism in HCC patients is poorly understood these days. In general, in patients with malignancies, tumor-secreted factors and/or host-tumor interactions result in an imbalance of energy demand and energy supply [37]. In our data, the difference of npRQ level between HCC and non-HCC patients was significant (*p* = 0.0074), suggesting the impact of HCC on energy metabolism. Significant correlation between npRQ and serum Zn level was found irrespective of the presence of HCC, supporting the usefulness and robustness of serum Zn level for the assessment of energy metabolism in CLDs.

The median serum Zn level was 64 μg/dL in this analysis. In LC patients, the proportion of patients with serum Zn level < 60 μg/dL was 54.4% (168/309) and in non-LC patients, that was 15.5% (43/277). Clinicians should be aware that a certain number of patients have hypozincemia even in non-LC patients. Regular follow-up for serum Zn level should be needed even in non-LC patients.

Several limitations should be acknowledged to the current analysis. Firstly, this study was a retrospective single-center study with cross-sectional analysis. Secondly, the number of patients with Child–Pugh C was small for analysis, although the strong point of the study was its large sample number (*n* = 586). Thirdly, the npRQ value can be affected by a patient’s daily activities or daily diet, leading to bias. Finally, although npRQ level was significantly correlated with serum Zn level for all cases and for all subgroups, the *r* values were not so impressive except for that between serum Zn level and Child–Pugh C. Caution must therefore be applied to the interpretation of our study data. Notwithstanding, our study results showed that npRQ level in CLDs was closely linked to serum Zn level.

## 5. Conclusions

In conclusion, serum Zn level can be a helpful marker for npRQ in patients with CLDs. Clinicians should be aware that serum Zn level can be an alternative for energy metabolism in patients with CLDs.

## Figures and Tables

**Table 1:** Baseline data (*n* = 586).

Parameters	Number or Median (Interquartile Range)
Gender, male/female	290/296
Age (years)	63 (54, 71)
Body mass index (kg/m^2^)	22.2 (20.2, 24.9)
Presence of LC, yes/no	309/277
Child–Pugh A, B and C (LC patients)	169/118/22
Presence of HCC, yes/no/unknown	161/422/3
Ascites, yes/no/unknown	50/533/3
Skeletal muscle mass index (kg/m^2^), male	7.3 (6.8, 8.0)
Skeletal muscle mass index (kg/m^2^), female	5.8 (5.3, 6.3)
ECW to TBW ratio	0.385 (0.376, 0.393)
Causes of liver disease Hepatitis B/Hepatitis C/alcohol/NAFLD or NASH/AIH or PBC/others	62/309/44/71/71/29
Liver histology F0/F1/F2/F3/F4	12/113/77/75/309
npRQ	0.86 (0.82, 0.93)
Total bilirubin (mg/dL)	0.9 (0.7, 1.3)
Serum albumin (g/dL)	3.8 (3.3, 4.1)
Prothrombin time (%)	86.0 (72, 95.4)
Platelets (×10^4^/mm^3^)	12.3 (7.8, 19.0)
Total cholesterol (mg/dl)	160 (135, 188)
AST (IU/L)	39.5 (28, 62)
ALT (IU/L)	35.5 (23, 62)
Serum zinc (μg/dL)	64 (55, 73)
HbA1c (NGSP)	5.2 (4.9, 5.7)
Branched-chain amino acid to tyrosine ratio	5.04 (3.73, 6.335)

LC; liver cirrhosis, HCC; hepatocellular carcinoma, ECW; extracellular water, TBW; total body water, NAFLD; non-alcoholic fatty liver disease, NASH; non-alcoholic steatohepatitis, AIH; autoimmune hepatitis, PBC; primary biliary cholangitis, npRQ; non-protein respiratory quotient, AST; aspartate aminotransferase, ALT; alanine aminotransferase, NGSP; National Glycohemoglobin Standardization Program.

**Table 2:** ROC analyses for npRQ < 0.85.

	AUC	Sensitivity (%)	Specificity (%)	Cutoff Value
Age	0.57	49.2	65.4	66.5
Body mass index	0.56	48.4	66.5	23.1
Serum zinc	0.69	62.2	60.7	60
Serum albumin	0.63	61.2	61.6	3.7
BTR	0.60	60.0	59.0	4.92
PT	0.65	56.3	68.5	81
Platelet count	0.60	47.9	53.9	9.3
Total bilirubin	0.59	41.7	73.8	1.2
Total cholesterol	0.58	63.7	52.3	164
HbA1c	0.53	26.0	87.5	4.7

AUC; area under the receiver operating characteristic curve, BTR; branched-chain amino acid to tyrosine ratio, PT; prothrombin time.
